# Physicochemical characteristics, antioxidant potential, and shelf stability of developed roselle–fig fruit bar

**DOI:** 10.1002/fsn3.3436

**Published:** 2023-05-16

**Authors:** Hunaina Aslam, Muhammad Nadeem, Urooj Shahid, Muhammad Modassar Ali Nawaz Ranjha, Waseem Khalid, Tahir Mahmood Qureshi, Muhammad Ather Nadeem, Alaiha Asif, Mehak Fatima, Muhammad Abdul Rahim, Chinaza Godswill Awuchi

**Affiliations:** ^1^ Institute of Food Science and Nutrition University of Sargodha Sargodha Pakistan; ^2^ Government General Hospital Ghulam Muhammad Abad Faisalabad Pakistan; ^3^ University Institute of Food Science and Technology The University of Lahore Lahore Pakistan; ^4^ Department of Food Sciences Cholistan University of Veterinary and Animal Sciences Bahawalpur Pakistan; ^5^ Department of Agronomy, College of Agriculture University of Sargodha Sargodha Pakistan; ^6^ Institute of Food Science and Nutrition Bahauddin Zakariya University Multan Pakistan; ^7^ Department of Food Science, Faculty of Life Sciences Government College University Faisalabad Punjab Pakistan; ^8^ School of Natural and Applied Sciences Kampala International University Kampala Uganda

**Keywords:** minerals, physicochemical characteristics, phytochemcials, roselle–fig fruit bar, sensory attributes, shelf stability

## Abstract

Fruit bars are prepared by combining different ingredients which are wholesome and nutrient rich. The current study was designed to develop roselle–fig (different proportions) fruit bars and further investigate their physicochemical characteristics and antioxidant potential. Moreover, the prepared fruit bars were scrutinized for microbial and sensory characteristics to assess the appropriateness of ingredients during storage (up to 90 days). It was observed that there was gradual increase in moisture content with the passage of time, while ash, fat, fiber, and protein contents did not change significantly during storage. The pH and total soluble solid contents of these fruit bars during storage were 3.54–4.07 and 1.71–1.86 Brix, respectively. According to the mean values for sensory evaluation, *T*_2_ was preferred over other treatments. The bars received an acceptable sensory evaluation, demonstrating that they were suitable for 90 days of storage. Phytochemical quantities significantly increased in subsequent treatments, but decreased during storage in all the treatments. Similar trend was observed regarding total antioxidant and DPPH radical scavenging activities. The mineral contents increased significantly among the treatments. The microbial analysis of fruit bars exposed that the fruit were microbiologically safe. Hence, on the basis of the results obtained in this study, it may be concluded that the developed roselle–fig fruit bar would be acceptable and nutritious regarding physicochemical characteristics, microbiological quality, and antioxidant potential.

## Graphical abstract

This study was designed to develop roselle–fig (different proportions) fruit bars and further investigate their physicochemical characteristics and antioxidant potential. Roselle–fig fruit bars are good source of proteins, minerals, fiber, and bioactive compounds which can be helpful to minimize nutritional deficiencies.

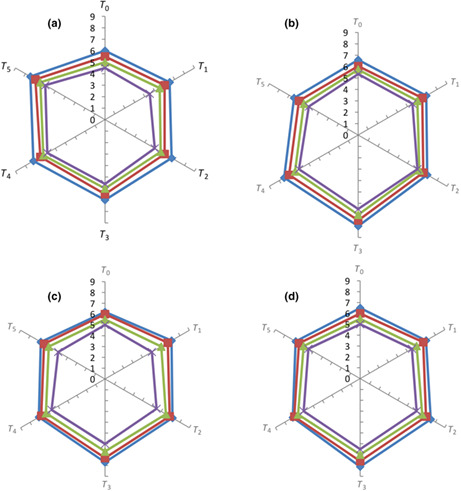

## 1 INTRODUCTION

Changes in the daily routine of a large portion of the world's population have resulted in an increase in demand for ready‐to‐eat convenience products (Popkin et al., 2020). Nowadays, the interest of people in food is not only to control hunger, but also to seek the products which could be beneficial for health or may reduce the chance of development of certain illnesses (Aja et al., 2023; Roberfroid, 2000; Santos et al., 2011). Usually people enjoy eating fresh fruits of the season, but there is a lack of their availability throughout the year. Therefore, it is imperative to preserve them in order to relish their nutrition throughout the year. Since many fruits contain majority of the flavoring and nutritional components, hence these can be used to create snack foods (Ukkuru & Pandey, 2007). The snack foods are relatively growing very fast as compared to other food products (Paiva, 2008). Fruit bars (to be used as snack food) may be developed in response to market demand for high‐quality nutritional food (Ryland et al., 2010). Such kinds of fruit bars are useful to provide nutrition and health benefits to the individuals of different ages. It has been reported that fruit bars are a type of processed food that offers nutritional value in the form of vitamins, minerals, and fiber (Parekh et al., 2014). It is considered as a nutrient‐dense diet (concerning bioactive compounds) which is required for the proper functioning of the body (Sun‐Waterhouse et al., 2010). The shelf life of fruit bars is generally long as compared to fresh fruits (Joshipura et al., 2001). Fruit bars are not only consumed by people for energy, but also for the gratification (Silva et al., 2018). Fruit bars are gaining popularity due to their excellent shelf life, flavor, texture, attractive packaging, and portability (Parimita & Arora, 2015). The healthy fruit bars would be better replacement of junk foods.

Roselle (*Hibiscus sabdariffa*) belongs to the Malvaceae family (Mahadevan & Kamboj, 2009) which is an underutilized plant in Pakistan with a lot of medicinal and nutritional properties (Abbas & Ali, 2011). It is broadly dispersed in tropical as well as subtropical regions and could be found in nearly all warm countries (Qi et al., 2005). The multipurpose roselle plant has a lot of importance in traditional medicines and has been reported to cure many diseases such as sores, ulcers, intoxication, and cough (Prasongwatana et al., 2008). Roselle has also been reported to cure cardiovascular problems, inflammatory illnesses, and cancer (Nkumah, 2015). Although its seeds, leaves, and calyces are considered valuable, the calyces offer higher nutritional and medicinal values. Roselle calyces are red in color (Cisse et al., 2009), sweet and tart in taste (Monteiro et al., 2017), and are good source of vitamin C, minerals, organic acids, fibers, and bioactive compounds like polyphenols (antioxidants), phytosterols, and organic acids (oxalic and succinic acid; Cisse et al., 2009). It has higher ascorbic acid level than orange and mango (Wong et al., 2003). It was also observed that 100 g of commercially available dried calyces contained 17 mg ascorbic acid, 150 mg calcium, 2 g protein, 10.2 g carbohydrates, and 3 mg iron (Al‐Ansary et al., 2016). Moreover, roselle calyx is also rich in pectin and may be used to make jam, jelly, wines, cakes, and pudding (as fresh). It is also used in making tea, sauces, ice creams, and other desserts (Emmy, 2006; Tsai et al., 2002). Some studies concerning roselle‐based drinks (Abidoye et al., 2022; Ehirim et al., 2022), fruit leathers, and jam (Fresua et al., 2022; Thonabut, 2021) have been carried out so far. Even though many other products can be prepared due to its unique taste, unfortunately it does not pique the interest of Pakistani food producers due to less awareness of its health benefits.

Fig (*Ficus carica*) fruit is sweet in taste and good source of minerals, vitamins, carbohydrates, organic acids, and phenolic compounds. Fig fruit has been reported to have appreciable levels of iron, calcium, magnesium, fibers (Michailides, 2003), polyphenols, and amino acids with negligible quantities of fat and cholesterol (Veberic et al., 2008: Crisosto et al., 2010). It is also reported that fig fruit can be used in the treatment of many illnesses such as diarrhea, loss of appetite, colic, cardio as well as respiratory (Duke, 2002), hepatoprotective (Gond & khadabadi, 2008), immune response (Yang et al., 2009).

Due to intense competition in the consumer market, industries are constantly in search of new ingredients that can enhance nutritional quality and add value to the final product (Awuchi et al., 2023; Ruiz‐Ojeda et al., 2019). Owing to the health benefits of roselle calyx and figs, it would be nice to produce fruit bars with more health benefits and minimal processing. However, combining these two fruits into fruit bars could be a lucrative business idea. Therefore, this study was focused to investigate the aptness of fig and dried roselle for use in nutrient‐dense fruit bars through physicochemical and sensory analyses. Moreover, shelf stability and antioxidant potential of such fruit bar were also monitored.

## 2 MATERIALS AND METHODS

### 2.1 Purchasing of raw materials

Dried calyces of roselle were purchased from Faisalabad (Pakistan), while the figs were purchased from the local market of Sargodha (Pakistan). Chemicals (Merck, Germany) for the analysis of final product were purchased from Merck distributor in Islamabad (Pakistan).

### 2.2 Preparation of basic ingredients and treatment plan

Roselle calyx and figs were obtained in dried form and weighed according to the ratio given in treatment plan. These were then washed under running tap water to remove dust/dirt or any adhering material. Further steaming was done in steamer to decrease the microbial load and to soften the figs and dried roselle. Before washing and steaming, dried figs contain 5.98% moisture and roselle 7.64% moisture. The steaming process also facilitates figs and roselle to convert them into paste by passing through mincing machine. The moisture content of fresh fruit bars ranged from 19.60% to 29.45%. Table 1 shows the proportions of each ingredient.

**TABLE 1:** Variable constituents of roselle–fig fruit bar (treatment plan).

Treatment	Roselle calyces (g)	Figs (g)
*T*_0_	0	100
*T*_1_	10	90
*T*_2_	20	80
*T*_3_	30	70
*T*_4_	40	60
*T*_5_	50	50

### 2.3 Development of roselle–fig fruit bar

Roselle–fig fruit bars were prepared by the method described by Nadeem et al. (2018) with some modifications. All the necessary machines and tools and surfaces were thoroughly cleaned with clear water and further contact surfaces were cleaned with ethanol and dried before starting any operation. Moreover, gloves were used for sheeting and cutting and handling of raw material. The steamed roselle calyces and figs were placed in mincing machine (Model: WF‐3250 R; WestPoint Electronics, Bordeaux, France) to form fruit paste. The fruit paste was moved to a sheeting and cutting table so that the bar shape could be more clearly achieved. After it had been sheeted using a roller, a cutter was used to cut the sheet into 2.5 cm wide × 1 cm thick × 7.5 cm long bars. The bar weighing approximately 25 g were sealed in airtight aluminum foil. These packs were stored at refrigerated temperature (4 ± 2°C).

### 2.4 Physicochemical analysis of roselle–fig fruit bar

Proximate analysis developed fruit bars, such as moisture, crude fiber (%), protein (%), fat (%), and ash (%), were scrutinized by the methods described in AOAC (2019). The pH was measured using a digital pH meter (Satorious, Germany; AOAC, 2019). Total soluble solids (TSS) were determined with the help of Atago Hand Refractometer by the method described previously (AOAC, 2019).

### 2.5 Evaluation of phytochemicals and antioxidant potential of roselle–fig fruit bar

#### 2.5.1 Sample preparation

For the evaluation of antioxidant potential, the samples were prepared by grinding 5 g of each bar to a fine powder with the help of a grinder. Then, 50 mL of the solvent was added into each sample to make homogeneous suspension. The mixture was then transferred into polypropylene tubes and shaken for approximately 1 h at room temperature. For proper extraction, the sample was filtered twice with Whatman filter paper after centrifugation at 1509.3 *g* for 10 min. The extract was stored at 4°C for later use.

#### 2.5.2 Determination of total phenolic (TP) contents

The Folin–Ciocalteu reagent method as explained by Salleh et al. (2017) was used for the determination of TP contents in all the samples. For the analysis, 400 μL of diluted samples were used for the reaction of 1 mL Folin–Ciocalteu reagent (10%) and 2 mL Na_2_CO_3_ (20%) in sequence. The absorbance of above mixture was measured at 760 nm using a spectrophotometer. Gallic acid was used as the standard and TP contents were expressed as mg of gallic acid equivalents (GAE) per 100 g.

#### 2.5.3 Determination of total flavonoid (TF) contents

The TF contents of roselle–fig fruit bar were assessed by the method described by Piskov et al. (2020). For analysis, 250 μL of diluted samples were taken before addition of 75 μL NaNO_2_ (5%), 150 μL AlCl_3_ (10%), and 500 μL NaOH (1 M) in sequence. Furthermore, 2 mL distilled water was also added into the above mixture. The absorbance was measured at a wavelength of 510 nm by using a spectrophotometer. Catechin (in ethanol) served as the standard and the TF contents were expressed as mg of (+) catechin equivalent (CE) per 100 g.

#### 2.5.4 Determination of total antioxidant activity (TAA)

The TAA of samples was calculated by applying a method described by Prieto et al. (1999). Four milliliter of the reagent (0.6 M H_2_SO_4_, 28 mM sodium phosphate, 4 mM ammonium molybdate) was added into 400 μL of each diluted sample. The absorbance was measured with a spectrophotometer at a wavelength of 695 nm. Trolox was used to create standard calibration curves. The TAA was measured in μg Trolox equivalent (TE) per gram of sample.

#### 2.5.5 Determination of DPPH radical scavenging activity

The method explained by Wang and Gao (2013) was used to determine DPPH radical scavenging activity of roselle–fig fruit bar. For the experiment, 1000 μL of the diluted sample was taken. The spectrophotometer was used for taking absorbance of all prepared samples at 517 nm. Ascorbic acid was used to create standard calibration curves. In this way, the DPPH radical scavenging activity was expressed as μmol ascorbic acid equivalents (AAE) per gram.

### 2.6 Mineral analysis of roselle–fig fruit bar

Mineral contents of roselle–fig fruit bar were assessed using atomic absorption spectrophotometer (AA‐Shimadzu Japan) by wet digestion method as described in AACC (2000).

### 2.7 Microbiological analysis of roselle–fig fruit bar

#### 2.7.1 Total plate counts (TPC)

By using method no. 42‐11 (AACC, 2000), TPC were determined. The fruit bar sample (each 1 g) in crushed form was placed in a sterile blender jar (having diluents of 9 mL buffered phosphate). The sample was blended at slow speed for 1–2 min. One milliliter of already available sample was transferred to 9 mL for preparation of decimal dilution. To resuspend material, the dilutions were agitated. One milliliter from each dilution was taken and placed on marked duplicate Petri dishes. About 12–15 mL of cooled plate count agar was placed on plates and allowed them to solidify. The dilutions were poured onto plates. To mix agar medium and sample dilutions, the plates were rotated. The inverted Petri dishes were incubated for about 48 h at 35**°**C. The colonies were counted and multiplied with dilution factor. The results were expressed in log_10_ CFU/g.

### 2.8 Sensory evaluation

The prepared fruit bars in this study were evaluated for sensory characteristics such as appearance and color, taste, texture, and overall acceptability by a panel of 20 members including faculty and postgraduate students from the Institute of Food Science and Nutrition, University of Sargodha, by following the 9‐point Hedonic scale (Santos et al., 2011).

### 2.9 Statistical analysis

Statistical analysis was performed using Minitab statistical software version 16 (Minitab, Inc., State College, PA, USA). Data was analyzed by two‐way ANOVA and Tukey's test for pairwise comparison (at the level of *p* < .05) .

## 3 RESULTS AND DISCUSSION

### 3.1 Physicochemical characteristics of roselle–fig fruit bar

The moisture (%), crude fat (%), protein (%), ash (%), fiber (%), and nitrogen free extract (NFE) of roselle–fig fruit bars varied significantly (*p* < .05) among treatments, while nonsignificant variations were observed during storage in most of the attributes regarding physicochemical characteristics (Table 2). The moisture contents were significantly (*p* < .05) increased during storage. The highest value of moisture contents was observed in treatment *T*_0_ (29.45), while the lowest was found in *T*_5_ (19.60). The increase in moisture might be due to absorption of moisture from surrounding environment during storage. The results of this study showed that the crude protein and crude fat contents differed significantly (*p* < .05) among treatments, but nonsignificant effect was observed during storage. The protein contents were the highest in *T*_5_ (9.93%) and the lowest in *T*_0_ (8.48%). The increasing trend of protein in subsequent treatments might be due to the presence of more quantities of proteins in roselle. Therefore, addition of more and more roselle in subsequent treatments might be the main reason of that increasing trend. The maximum and minimum values for fat contents of roselle–fig fruit bar were observed in *T*_0_ (0.60%) and *T*_5_ (0.42%), respectively. On the contrary to protein, the decreasing trend of fat in subsequent treatments might be due to the presence of less quantities of fat in roselle. Therefore, addition of more and more roselle in subsequent treatments might be the main reason of that decreasing trend. The highest values of fiber contents were observed in *T*_5_ (4.10%) and the lowest in *T*_0_ (2.12%). The increasing trend of fiber in subsequent treatments might be due to the presence of more quantities of fibers in roselle. Therefore, addition of more and more roselle in subsequent treatments might be the main reason of that increasing trend. A nonsignificant effect of treatment and storage on ash contents of roselle–fig fruit bars was observed. The increasing trend of ash in subsequent treatments might be due to the presence of more quantities of minerals in roselle. Therefore, addition of more and more roselle in subsequent treatments might be the main reason of that increasing trend. The NFE usually depicts the estimated quantities of water‐soluble polysaccharides (sugars, starch) in a sample. The lowest NFE was observed in *T*_0_ (56.74%) and the highest was recorded in *T*_5_ (60.83%). The increasing trend of NFE in subsequent treatments might be due to the presence of more quantities of water‐soluble polysaccharides in roselle. There is a significant effect of storage on NFE of roselle–fig fruit bars. The results regarding the change in proximate composition were in close agreement to the findings of Munir et al. (2016) (in date bars) and Nadeem et al. (2012) (in apricot–date bars), who observed that moisture, crude protein, crude fat, and crude fiber contents varied significantly among treatments. Similar results were also reported by Ahmad et al. (2005).

**TABLE 2:** Physicochemical characteristics of roselle–fig fruit bar.

Components	Days	Treatments					
		*T*_0_	*T*_1_	*T*_2_	*T*_3_	*T*_4_	*T*_5_
Moisture	0	29.45 ± 1.04	26.83 ± 1.04	24.43 ± 1.09	22.60 ± 1.03	21.85 ± 1.03	19.60 ± 1.05
	90	32.70 ± 1.03	29.40 ± 1.01	28.60 ± 1.02	28.40 ± 1.03	26.80 ± 1.03	26.20 ± 1.03
Ash	0	2.62 ± 0.03	3.11 ± 0.03	3.61 ± 0.04	4.11 ± 0.02	4.63 ± 0.03	5.12 ± 0.03
	90	2.59 ± 0.03	3.10 ± 0.02	3.59 ± 0.03	4.12 ± 0.02	4.63 ± 0.01	5.11 ± 0.02
Fat	0	0.60 ± 0.02	0.57 ± 0.02	0.52 ± 0.02	0.49 ± 0.03	0.45 ± 0.02	0.42 ± 0.05
	90	0.61 ± 0.02	0.57 ± 0.01	0.51 ± 0.02	0.49 ± 0.02	0.46 ± 0.02	0.42 ± 0.03
Fiber	0	2.12 ± 0.03	2.51 ± 0.03	2.91 ± 0.03	3.31 ± 0.03	3.70 ± 0.03	4.10 ± 0.03
	90	2.10 ± 0.03	2.49 ± 0.02	2.92 ± 0.02	3.3 ± 0.03	3.71 ± 0.02	4.09 ± 0.03
Protein	0	8.48 ± 0.03	8.77 ± 0.02	9.07 ± 0.02	9.35 ± 0.03	9.65 ± 0.03	9.93 ± 0.02
	90	8.47 ± 0.03	8.74 ± 0.02	9.05 ± 0.02	9.33 ± 0.03	9.62 ± 0.03	9.92 ± 0.02
NFE	0	56.74 ± 1.09	58.21 ± 1.03	59.46 ± 1.06	60.08 ± 1.07	59.52 ± 1.30	60.83 ± 1.09
	90	53.52 ± 1.11	55.70 ± 1.01	55.33 ± 1.10	54.35 ± 1.02	54.78 ± 1.10	54.27 ± 1.09
pH	0	5.23 ± 0.03	3.86 ± 0.03	3.30 ± 0.01	3.06 ± 0.03	2.96 ± 0.03	2.86 ± 0.03
	90	6.14 ± 0.01	4.84 ± 0.03	3.77 ± 0.02	3.42 ± 0.03	3.18 ± 0.02	3.05 ± 0.01
TSS	0	4.05 ± 0.02	2.03 ± 0.02	1.03 ± 0.03	1.05 ± 0.05	1.04 ± 0.04	1.07 ± 0.03
	90	4.98 ± 0.03	2.01 ± 0.01	1.08 ± 0.01	1.05 ± 0.01	1.02 ± 0.01	1.01 ± 0.01

*Note*: *T*_0_ = (control) roselle–fig fruit bar having 100% fig; *T*_1_ = roselle–fig fruit bar having 10% roselle and 90% fig; *T*_2_ = roselle–fig fruit bar having 20% roselle and 80% fig; *T*_3_ = roselle–fig fruit bar having 30% roselle and 70% fig; *T*_4_ = roselle–fig fruit bar having 40% roselle and 60% fig; *T*_5_ = roselle–fig fruit bar having 50% roselle and 50% fig.

The pH of fruit bars containing varying amounts of roselle and fig differed significantly (*p* < .05) between treatments (Table 2). The maximum pH was observed in treatment *T*_0_ (5.23) and the minimum was observed in treatment *T*_5_ (2.86), because the pH of fig is more as compare to roselle. The pH of roselle–fig fruit bar increased significantly during storage (0–90 days) due to addition of less acidic contents from fig. The pH value expresses the intensity of the acidity of a food. It is one of the vital factors that determine the microbial growth during food processing and storage. A similar trend of pH was reported by Ranjha et al. (2020). They documented that the control date bar had higher pH value as compared to the date bar having 3% PPE (pomegranate peel extract). Similar outcomes were observed by Kumar et al. (2017) in papaya–guava fruit bar. It was observed that the pH of the papaya–guava fruit bar increased from 3.58 to 3.61 after 60 days of storage, possibly because of the generation of free acids and the hydrolysis of pectin (Imran et al., 2000). Durrani et al. (2010) and Vidhya and Narain (2011) obtained same outcomes for mango pulp and wood apple bar, respectively. Moreover, Bilginer (2019) prepared beverage from roselle and noticed an increase in pH value during storage period (up to 4 months).

The effect of treatment and storage on total soluble solids (°Brix) of roselle–fig fruit bar is presented in Table 2. The mean values of TSS within treatments ranged from 4.51°Brix to 1.04°Brix. The highest TSS value was observed in *T*_0_ (4.51°Brix) and the lowest value was found in *T*_5_ (1.04°Brix). The mean values of TSS of roselle–fig fruit bar during storage indicated that TSS of all samples did not vary significantly in treatments (*T*_1_ to *T*_5_) during storage from 0 to 90 days.

### 3.2 Total phenolic (TP) contents

**FIGURE 1:**
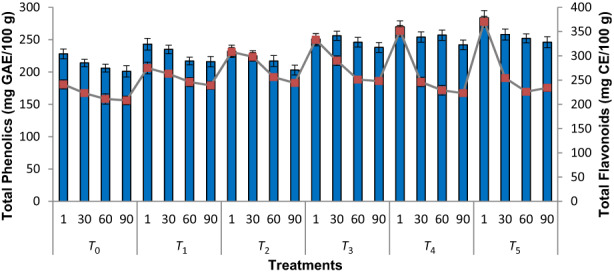
Phytochemicals (total phenolics and flavonoids, mean values ± SD) of roselle–fig fruit bars during storage. *T*_0_ = (control) roselle–fig fruit bar having 100% fig; *T*_1_ = roselle–fig fruit bar having 10% roselle and 90% fig; *T*_2_ = roselle–fig fruit bar having 20% roselle and 80% fig; *T*_3_ = roselle–fig fruit bar having 30% roselle and 70% fig; *T*_4_ = roselle–fig fruit bar having 40% roselle and 60% fig; *T*_5_ = roselle–fig fruit bar having 50% roselle and 50% fig.

Figure 1 shows the mean values for TP contents of roselle–fig fruit bars (treatments) during storage (90 days) at refrigerated temperature. There was significant influence of roselle addition in fig bar on TP contents between treatments and storage. The TP contents in all the fruit bars decreased with the increase of storage time. The TP contents were found the highest (284.22 mg GAE/100 g) in fresh fruit bars (*T*_5_), whereas the lowest (228.52 mg GAE/100 g) were observed in freshly prepared control treatment (*T*_0_). The decreasing trend might be attributed to the oxidation of phenolics due to higher activity of naturally present polyphenol oxidases in figs. By increasing the quantities of roselle calyces in the successive treatments (fruit bars), TP contents also increased. Our results were accordant with the findings of Shin et al. (2021) who observed increased total polyphenol contents in yogurt (treatments) by the gradual increase in dried roselle calyx into their formulations.

The higher the phenolic compounds, the higher the antioxidant activity (Miladi & Damak, 2008; Zeghoud et al., 2023). The trend of total phenolic contents observed in this study were similar to that observed by Ranjha et al. (2020), who prepared date bars fortified with APE (apple peel extract) and PPE (pomegranate peel extract). In addition, our results of this study were also very similar to the findings of Nadeem et al. (2012), who reported that the addition of dried apricot paste caused an increase in the phenolic content of fruit bars. The treatment containing the lowest quantity of dried apricot paste had the lowest phenolic contents, while the treatment containing the highest quantity of dried apricot paste showed the maximum values. Current findings are consistent with the earlier observations of Pawar et al. (2017), who found a significant decrease in total polyphenols of cocoa–mulhati guava bar during 90 days. Even though there were appreciable quantities of TP contents in *T*_0_ (comprising 100% fig fruit), it was also observed that there was slight increment in TP contents in successive treatments. Such kind of slight variations in the treatments might be attributed to the addition of more and more roselle calyces in subsequent treatments (bars). This showed that roselle calyces also contained significant quantities of TP contents.

#### 3.2.1 Total flavonoid (TF) contents

The mean values for TF contents of roselle–fig fruit bars (treatments) during storage (90 days) at refrigerated temperature are presented in Figure 1. There was significant (*p* ≤ .05) influence of roselle calyces on TF contents of fruit bars during storage. The TF contents in all the fruit bars decreased with the increase of storage time which might be due to degradation of flavonoids with the passage of time. The TF contents were found the highest (370.26 mg CE/100 g) in freshly prepared fruit bars during *T*_5_ treatment, whereas the lowest (241.42 mg CE/100 g) were observed in freshly prepared fruit bars during *T*_0_ treatment.

Yen et al. (2022) investigated the red dragon fruit bar and found similar results. As the concentration of passion fruit increases, so the flavonoids contents increased. The flavonoids from the passion fruit were thought to be the cause of the higher TP and TF levels in the fruit bar. Moreover, the passion fruit also had ascorbic acid, which protected the flavonoids in the fruit bar. Kolniak‐Ostek et al. (2013) found that adding ascorbic acid to pasteurized cloudy apple juices had a positive effect on the polyphenol compounds. Similarly, the outcomes are consistent to the findings of Nadeem et al. (2012) who observed a gradual decrease in flavonoid content during storage of 120 days. At the day of production, the total flavonoids contents were 106.63 mg CE/g, which decreased to 44.91 mg CE/g after 120 days. Free radicals are also produced to a greater extent during storage, which may also reduce the total flavonoids contents. Del Caro et al. (2004) prepared orange juice and stored at 4°C and showed decrease in flavonoid contents during storage.

Even though there were appreciable quantities of TF contents in *T*_0_ (comprising 100% fig fruit), it was also observed that there was slight increment in TF contents in successive treatments. Such kind of slight variations in the treatments might be attributed to the addition of more and more roselle calyces in subsequent treatments (bars). This showed that roselle calyces also contained significant quantities of TF contents. Our results were accordant with the findings of Shin et al. (2021) who observed increased total flavonoid contents in yogurt (treatments) by the gradual increase in dried roselle calyx into their formulations.

### 3.3 Total antioxidant activity (TAA)

**FIGURE 2:**
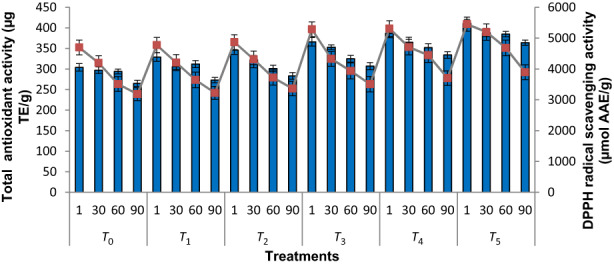
Total antioxidant activity (TAA, mean values ± SD) and DPPH radical scavenging activity (mean values ± SD) of roselle–fig fruit bars during storage. *T*_0_ = (control) roselle–fig fruit bar having 100% fig; *T*_1_ = roselle–fig fruit bar having 10% roselle and 90% fig; *T*_2_ = roselle–fig fruit bar having 20% roselle and 80% fig; *T*_3_ = roselle–fig fruit bar having 30% roselle and 70% fig; *T*_4_ = roselle–fig fruit bar having 40% roselle and 60% fig; *T*_5_ = roselle–fig fruit bar having 50% roselle and 50% fig.

The mean values for TAA of roselle–fig fruit bars (treatments) during storage (90 days) at refrigerated temperature are presented in Figure 2. There was significant (*p* ≤ .05) influence of roselle calyces on TAA of fruit bars during storage. The TAA in all the fruit bars (treatments) decreased with the increase of storage time. The highest TAA (411.21 μg TE/g) were found in fresh fruit bars (*T*_5_), whereas the lowest (304.29 μg TE/g) were observed in control treatment (*T*_0_). By increasing the quantities of roselle calyces in the successive treatments (fruit bars), the TAA also increased. Even though there was notable values of TAA in *T*_0_ (comprising 100% fig fruit), it was also observed that there was slight increment in TAA contents in successive treatments. Such kind of slight variations in the treatments might be attributed to the addition of more and more roselle calyces in subsequent treatments (bars). This showed that roselle calyces contained enormous quantities of TP and TF contents as reported by a study who observed considerable quantities of total polyphenols (740 mg/100 g), flavonoids (350 mg/100 g), and anthocyanins (1653 mg/100 g) in roselle extract (Pacôme et al., 2014). During storage, phenolic as well as flavonoid contents might be degraded, resulting in a reduction of total antioxidant activity of fruit bars.

These outcomes were in agreement to the findings of Agrahari et al. (2004), who found decreasing trend of antioxidant activity of soy‐enriched apple bar during storage. They observed that the soy‐enriched apple bar contained 1.6 times more antioxidant activity than the regular apple bar. Sakhale et al. (2015) showed the similar results in jam prepared from fig. Many studies indicated that the antioxidant content of fruits decreased during storage. These results were comparable to those previously published by Kim and Padilla‐Zakour (2004), who revealed that the antioxidant capacity of plums, cherries, and raspberries decreased during storage. They further reported that the main reason for the decrement of antioxidant activity might be the degradation of anthocyanins. During storage, there was a decrease in bioactive compounds. The decreases in total phenolics, total flavonoids, and total anthocyanin were also observed in red jam (Zafrilla et al., 2001).

#### 3.3.1 DPPH radical scavenging activity

The mean values of DPPH radical scavenging activity of roselle–fig fruit bars (treatments) during storage (90 days) at refrigerated temperature are presented in Figure 2. Wu et al. (2018) investigated roselle extract for its anthocyanins and DPPH radical scavenging activity. They reported that the roselle extract was rich in anthocyanins and had good DPPH antioxidant capacity (DPPH IC_50_ = 4.06 mg/mL, >80% inhibition). There was significant (*p* ≤ .05) influence of roselle calyces on DPPH radical scavenging activity of fruit bars during storage. The DPPH radical scavenging activity in all the fruit bars (treatments) decreased with the increase of storage time. The highest DPPH radical scavenging activity (5454.61 μmol AAE/g) was found in fresh fruit bars (*T*_5_), whereas the lowest (4697.11 μmol AAE/g) was observed in control treatment (*T*_0_). By increasing the quantities of roselle calyces in the successive treatments (fruit bars), the DPPH radical scavenging activity also increased. Our results were in accordance with the findings of Shin et al. (2021), who observed increased antioxidant activity (depicted from IC_50_ values concerning DPPH radical scavenging activity) by the gradual increase in dried roselle calyx into yogurt formulations (treatments).

Considering the impact of treatment, these findings are similar to those reported by Yen et al. (2022), who observed increased DPPH radical scavenging activity of the red dragon fruit bar with increasing the levels of passion fruit. Similar results were found by Srivastava et al. (2019) in orange–guava fruit bar. They stated that the fruit bar having higher quantity of guava had the highest amount of ascorbic acid, while the fruit bar having less quantity of guava had the lowest amount of ascorbic acid. The higher TP and TF levels in the fruit bar containing roselle calyx resulted in greater DPPH radical scavenging activity. It was also reported that the increased antioxidant activity might be due to the presence of increased levels of ascorbic acid, betacyanins, and total phenolic and flavonoid contents in dragon fruit bar (with the addition of different levels of passion fruit; Yen et al., 2022). Most likely, this is because of the synergistic effects of these antioxidants, which has a high capacity to donate hydrogen atoms and thus neutralize the free radicals produced during the thermally induced oxidation and auto‐oxidation processes (Yen et al., 2022). Similar results were stated by Sakhale et al. (2015) in fig jam. They observed that at the day of production, the DPPH content were 43.70, which reduced to 28.90 after 60 days of storage at low temperature. Nevertheless, it may be assumed that the oxidative reaction and prolonged storage may affect hydrolysis of compounds and lead to gradual reduction. Ascorbic acid is extremely susceptible to heat. Therefore, thermal deterioration of ascorbic acid during manufacturing and oxidation may contribute to that decrease (Mutkule et al., 2014).

Even though there was notable values of DPPH radical scavenging activity in *T*_0_ (comprising 100% fig fruit), it was also observed that there was slight increment in DPPH radical scavenging activity in successive treatments. Such kind of slight variations in the treatments might be attributed to the addition of more and more roselle calyces in subsequent treatments (bars). This showed that roselle calyces contained enormous quantities of TP and TF contents which might have shown DPPH radical scavenging activity. During storage, phenolic as well as flavonoids might be degraded, thereby resulting in a reduction of DPPH radical scavenging activity of fruit bars.

### 3.4 Mineral contents of roselle–fig fruit bar

**FIGURE 3:**
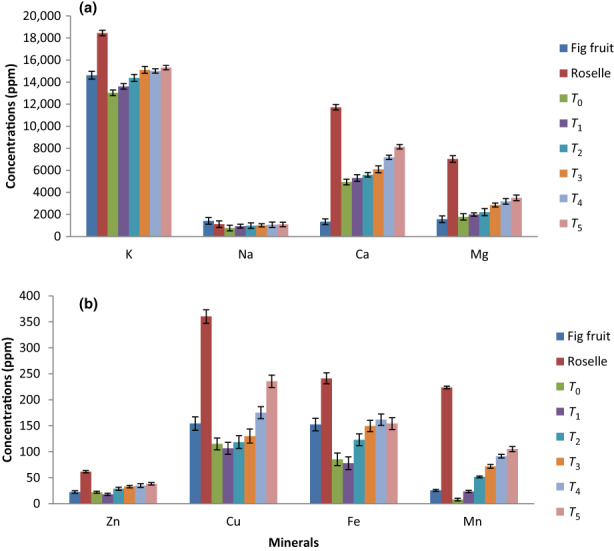
Mineral (K, Na, Ca, Mg [a] and Zn, Cu, Fe, Mn [b]) contents (ppm) of fig fruit, dried roselle calyces, and roselle–fig fruit bars. *T*_0_ = (control) roselle–fig fruit bar having 100% fig; *T*_1_ = roselle–fig fruit bar having 10% roselle and 90% fig; *T*_2_ = roselle–fig fruit bar having 20% roselle and 80% fig; *T*_3_ = roselle–fig fruit bar having 30% roselle and 70% fig; *T*_4_ = roselle–fig fruit bar having 40% roselle and 60% fig; *T*_5_ = roselle–fig fruit bar having 50% roselle and 50% fig.

The mean values regarding mineral composition of fruit bar are presented in Figure 3. In general, dried roselle contained very high quantities of potassium (K), magnesium (Mg), calcium (Ca), copper (Cu), iron (Fe), manganese (Mn), and zinc (Zn) as compared to fig fruit, while fig fruit contained higher content of sodium (Na) than roselle. Our results showed that mineral contents of fruit bars differed significantly (*p* ≤ .05). All the treatments (*T*_0_–*T*_5_; fruit bars) showed increasing trend of minerals as most of the minerals in dried roselle were found in higher concentrations. Among the major minerals, *K* was found in the highest concentration (18,449.88 ppm) in roselle calyx. In this way, K showed increasing trend in all the treatments. Similarly, Ca (11,720.40 ppm) and Mg (7022.47 ppm) were found in higher concentrations in roselle calyx compared to fig fruit, but Na (1111.18 ppm) was found in lower concentrations than fig fruit (1416.75 ppm). The minerals which were present in minute quantities included Zn (21.97–38.32 ppm), Cu (115.03–235.46 ppm), Fe (85.47–154.21 ppm), and Mn (8.12–105.04 ppm). It is evident from the results that the addition of roselle among treatments caused a significant increase in mineral contents of fruit bars. Therefore, these fruit bars may be recommended to treat human micronutrient deficiencies. These findings were in close agreement to the results of Munir et al. (2019), who observed an increase in mineral content of apricot bar as the concentration of ash increased in bars. El‐Sayed (2019) investigated minerals in roselle leaves. They found that the concentrations of K, Na, Ca, Mg, Zn, Fe, and Mn were 7940 (ppm), 711 (ppm), 13,500 (ppm), 4789 (ppm), 52 (ppm), 148 (ppm), and 70 (ppm), respectively. In this way, our results were in agreement to the findings of El‐Sayed (2019). Similar results were obtained by Jung et al. (2013) for roselle extract. They found that the concentrations of Ca, Na, Mn, Fe, and Zn contents were 10,707 (ppm), 450 (ppm), 253 (ppm), 163 (ppm), and 32 (ppm), respectively. The little bit discrepancies in the mineral contents of roselle extract might be due to variations in agro‐climatic variations.

### 3.5 TPC of roselle–fig fruit bar

At refrigerated temperature, mean values for TPC of roselle–fig fruit bar have been given in Table 3. The findings showed that TPC had significant (*p* ≤ .05) effect on treatments and storage. The mean values (log_10_ CFU/g) of TPC of *T*_0_ were the highest at all stages of storage period as compared to the counts of all other treatments, whereas the lowest counts were found in *T*_5_. It was observed that by increasing the concentrations of roselle calyces in the treatments, TPC gradually decreased as roselle had antimicrobial properties. During a 3‐month storage period at refrigerated temperatures, TPC grows significantly, but remains within tolerable limits. This rise in TPC may be attributable to the availability of nutrients in fruit bars.

**TABLE 3:** Total plate counts (log_10_ CFU/g; mean values ± SD) roselle–fig fruit bar during storage.

Treatments	Storage (days)			
	1	30	60	90
*T*_0_	2.02 ± 0.01^a–e^	2.05 ± 0.01^ab^	2.06 ± 0.01^ab^	2.07 ± 0.01^a^
*T*_1_	1.95 ± 0.07^f–j^	2.00 ± 0.01^b–g^	2.03 ± 0.01^a–d^	2.04 ± 0.01^a–c^
*T*_2_	1.92 ± 0.02^h–k^	1.97 ± 0.01^c–h^	2.00 ± 0.02^b–g^	2.01 ± 0.01^a–f^
*T*_3_	1.88 ± 0.02^j–m^	1.96 ± 0.02^d–i^	1.98 ± 0.01^c–h^	1.99 ± 0.01^b–g^
*T*_4_	1.83 ± 0.03^lm^	1.92 ± 0.02^h–k^	1.94 ± 0.02^g–k^	1.95 ± 0.02^e–i^
*T*_5_	1.68 ± 0.03^n^	1.81 ± 0.02^m^	1.87 ± 0.02^k–m^	1.90 ± 0.02^i–l^

*Note*: *T*_0_ = (control) roselle–fig fruit bar having 100% fig; *T*_1_ = roselle–fig fruit bar having 10% roselle and 90% fig; *T*_2_ = roselle–fig fruit bar having 20% roselle and 80% fig; *T*_3_ = roselle–fig fruit bar having 30% roselle and 70% fig; *T*_4_ = roselle–fig fruit bar having 40% roselle and 60% fig; *T*_5_ = roselle–fig fruit bar having 50% roselle and 50% fig.

The observations are in conformance with those obtained by Munir et al. (2019), who reported the same decreasing tendency among the treatment of apricot‐based bars, as the mean values varied from 3.0 log_10_ CFU/g (*T*_0_) to 2.2 log_10_ CFU/g (*T*_3_). The findings of this study were also supported by the earlier findings of Al‐Hooti, Sidhu, Al‐Otaibi, Al‐Ameeri, and Al‐Qabazard (1997), who discovered that TPC in date bar samples ranged from 1.00 to 2.18 log_10_ CFU/g. The change in TPC during storage for different fruit bars are in accordance to the results of Akhtar et al. (2014), who reported that TPC increased significantly in apple–date fruit bar during storage of 90 days from 0.5 log_10_ CFU/g to 3.21 log_10_ CFU/g. Similarly increase in TPC was observed by Nadeem et al. (2022) in water chestnut‐supplemented date bars. The increase in TPC during storage of 90 days ranged from 1.64 log_10_ CFU/g to 2.31 log_10_ CFU/g. The antioxidants present in both fig and roselle calyx may have effect on the shelf stability of prepared bars in this study.

### 3.6 Sensory characteristics of roselle–fig fruit bar

**FIGURE 4:**
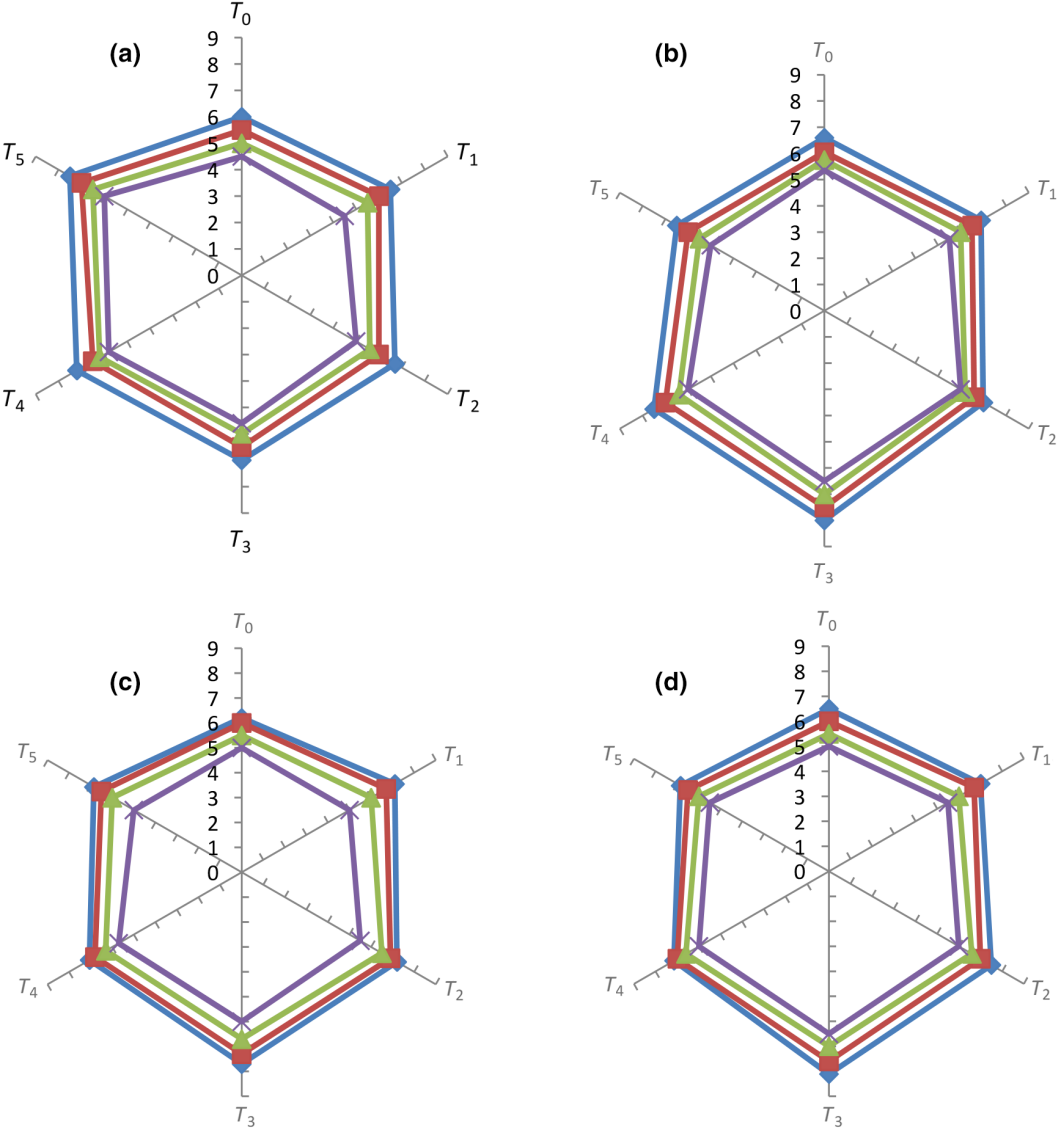
Sensory scores (appearance and color [a], taste [b], texture [c], and overall acceptability [d]) of roselle–fig fruit bars at 0 (blue bar), 30 (red bar), 60 (green bar), and 90 (purple bar) days of storage. *T*_0_ = (control) roselle–fig fruit bar having 100% fig; *T*_1_ = roselle–fig fruit bar having 10% roselle and 90% fig; *T*_2_ = roselle–fig fruit bar having 20% roselle and 80% fig; *T*_3_ = roselle–fig fruit bar having 30% roselle and 70% fig; *T*_4_ = roselle–fig fruit bar having 40% roselle and 60% fig; *T*_5_ = roselle–fig fruit bar having 50% roselle and 50% fig.

The fruit bars prepared in this study were assessed for appearance and color, taste, texture, and overall acceptability during storage period (0–90 days; Figure 4). The findings of this study revealed that the scores regarding appearance and color attribute among different treatments varied significantly (*p* < .05). Among the treatments, freshly prepared *T*_5_ showed the highest score (7.5), whereas freshly prepared *T*_0_ showed the lowest score (6.1). The color of roselle calyces was very appealing after addition into fruit bar as most of the people liked the color of fruit bar with the subsequent addition of roselle calyces. The color score of roselle–fig fruit bars was significantly (*p* < .05) affected by the storage period. The highest score for appearance and color was achieved by fresh bars which gradually decreased with the passage of time due to loss of their freshness. Color is the most important characteristic as it is the first impression perceived by the eyes of consumers and acceptability of product is highly affected by this characteristic. Discoloration in fruit bar may affect the impression of food. The panelists observed the darkness or increase in brown color during storage. Nonenzymatic browning during storage may cause decreased score regarding color. The increase in darkness might also be attributed to occurrence of Maillard reaction (Agrahari et al., 2004). Agrahari et al. (2004) observed the decreasing order of color score of soy‐enriched apple bar from 4.9 to 3.8 during period of 6 months storage. Similarly, Al‐Hooti, Sidhu, Al‐Otaibi, Al‐Ameeri, and Al‐Qabazard (1997) also found the decrease in color of date bars from 6.8 to 6.2 during storage period up to 6 months. Ahmed and Ramaswamy (2005) also observed that color of papaya fruit bars was affected significantly by storage periods. They explained that score of color varied from 6.6 to 6.2 in 6 months of storage period. Nadeem et al. (2018) also observed decrease in color score of date bars during storage up to 90 days. Nadeem et al. (2022) observed the similar results in water chestnut‐supplemented date bars.

It was observed that taste score varied significantly (*p* < .05) due to more addition of roselle calyces. The highest taste score (8.1) was obtained by freshly prepared *T*_3_ (roselle–fig fruit bar having 30% roselle and 70% fig), whereas the lowest score (6.5) was received by freshly prepared *T*_5_ as slight tartness increased after subsequent addition of roselle calyces. Nevertheless, all the treatments were acceptable regarding taste attribute. The taste score of roselle–fig fruit bars was significantly (*p* < .05) affected by the storage period. The highest score for taste was achieved by fresh bars which gradually decreased with the passage of time due to loss of their freshness.

Taste is perceived by the taste buds of the tongue, while the composition of food, flavor, and texture can modify it. A food is generally accepted or rejected by taste. The taste score was declined during storage because of development of bitterness with increased storage time (Sharma et al., 2013). Similar trend of taste score was observed in wood apple bar (Bhatt & Jha, 2015), choco–quinoa nutri bar (Padmashree et al., 2018), nutri bar (Athira et al., 2018), defatted soy flour cereal bar (Yadav & Bhatnagar, 2016), peach–soy fruit leather (Anju et al., 2014). Nadeem et al. (2012) found that the taste of date bars remained acceptable for the storage period up to 90 days, however, the score for taste decreased with increase in storage period. Prasad (2009) concluded that scores for taste of mango bars increased with the increase in quantity of mango fruit powder up to 30% and decreased thereafter. Nadeem et al. (2022) prepared a fruit bar supplemented with water chestnut and observed similar results. The highest taste score 7.72 was noticed at the production day, while the lowest taste score 5.79 was noticed after 90 days of storage.

Regarding texture, it was observed that the score varied significantly (*p* < .05) due to more addition of roselle calyces. The highest texture score (7.7) was obtained by freshly prepared *T*_3_ (roselle–fig fruit bar having 30% roselle and 70% fig), whereas the lowest score (6.2) was received by freshly prepared *T*_0_. The texture score of roselle–fig fruit bars was significantly (*p* < .05) affected by the storage period. The highest score for texture was achieved by fresh bars which gradually decreased with the passage of time due to loss of their freshness.

Texture is the most significant property which defines the consumer acceptability and overall quality of a food product. Texture can be scored by touch and visually. Texture is the perception of the rheological and structural characteristics of a product that can be judged by mechanical, tactile, visual, and auditory receivers. Jan et al. (2012) described a gradual reduction in the texture score (8.00 to 7.10) of nutri bars for lactating women. Silva et al. (2016) showed that texture values nonsignificantly decreased from 7.14 to 7.11 in bars made with Jeriva fruit flour in Brazil. Al‐Hooti, Sidhu, Al‐Otaibi, Al‐Ameeri, and Al‐Qabazard (1997) found that the texture score of date bars decreased from 7.0 to 5.9 during 1 to 180 days of storage. The changes in texture score of various treatments prepared in this study was also in accordance to the findings of Nadeem et al. (2022), who observed the highest texture score (7.53) in fresh water chestnut‐supplemented date bars, which gradually decreased to 6.00 after 90 days of storage. Reduction in texture score of fruit bars during storage may be attributed to hardening of fruit bar as a result of moisture loss during storage period (Aggarwal et al., 2022). Same decline was reported in defatted soy flour cereal bar (Yadav & Bhatnagar, 2016), choco–quinoa nutri bar (Padmashree et al., 2018), tomato and papaya fruit bar (Ahmed & Ramaswamy, 2005), and nutri bar (Athira et al., 2018).

The results regarding overall acceptability score of fruit bars showed significant (*p* < .05) effect of storage and treatments. It was observed that overall acceptability of fruit bars varied significantly (*p* < .05) due to more addition of roselle calyces. The highest overall acceptability score (8.11) was obtained by freshly prepared *T*_3_ (roselle–fig fruit bar having 30% roselle and 70% fig), whereas the lowest score (6.5) was received by freshly prepared *T*_0_. The overall acceptability score of roselle–fig fruit bars was significantly (*p* < .05) affected by the storage period. The highest score for overall acceptability was achieved by fresh bars which gradually decreased with the passage of time due to loss of their freshness.

Sensory characteristics generally indicate the whole sensory quality of foods. The decreasing trend of scores concerning different sensory attributes of different fruit bars has also been observed in many other studies (Al‐Hooti, Sidhu, Al‐Otaibi, Al‐Ameeri, & Al‐Qabazard, 1997; Al‐Hooti, Sidhu, Al‐Otaibi, Al‐Ameeri, & Qabazard, 1997; Parekh et al., 2014; Vijayanand et al., 2000).

Studies revealed that roselle and fig are appropriate for the quality production of fruit bars having high nutritional value and health benefits. Roselle–fig fruit bars are good source of proteins, minerals, fiber, and bioactive compounds which can be helpful to minimize nutritional deficiencies. Thus, fruit bar development and characterization through different parameters is good to increase its nutritional value. Such bars can be eaten at any time which can fulfill the nutritional requirements of all age groups. Consequently, it might have a lot of sales potential due to its high nutritional value.

## 4 CONCLUSION AND RECOMMENDATIONS

The results revealed that there was significant effect on the ash, fat, fiber, and protein contents of developed bars by the addition of roselle calyx in the formulation of fig bar. The total phenolic and flavonoid contents significantly increased in subsequent treatments, but decreased during storage in all the treatments. Similar trend was observed regarding total antioxidant and DPPH radical scavenging activities. The mineral contents increased significantly among the treatments. The microbial analysis of fruit bars exposed that the fruit were microbiologically safe. According to the mean values for sensory evaluation, *T*_2_ was preferred over other treatments. The bars received an acceptable sensory evaluation, demonstrating that they were suitable up to 90 days of storage. On the basis of the findings of this study, it may be concluded that the developed bar would be nutritious owing to the presence of benefits of fig and roselle.

Thus, it may be recommended that roselle–fig fruit bar should be manufactured at industrial scale due to their high nutritional value, delicious taste, and attractive color. Such kind of bar can be utilized as nutraceutical and functional foods.

## AUTHOR CONTRIBUTIONS

**Hunaina Aslam:** Conceptualization (equal); data curation (equal); formal analysis (equal); investigation (equal); methodology (equal); project administration (equal); resources (equal); software (equal); validation (equal); writing – original draft (equal). **Muhammad Nadeem:** Conceptualization (equal); data curation (equal); formal analysis (equal); methodology (equal); project administration (equal); supervision (equal); visualization (equal); writing – review and editing (equal). **Urooj Shahid:** Conceptualization (equal); data curation (equal); formal analysis (equal); investigation (equal); project administration (equal); software (equal); supervision (equal); writing – original draft (equal); writing – review and editing (equal). **Muhammad Modassar Ali Nawaz Ranjha:** Data curation (equal); formal analysis (equal); investigation (equal); project administration (equal); software (equal); visualization (equal); writing – original draft (equal); writing – review and editing (equal). **Waseem Khalid:** Data curation (equal); investigation (equal); methodology (equal); project administration (equal); resources (equal); software (equal); validation (equal); writing – original draft (equal). **Tahir Mahmood Qureshi:** Data curation (equal); investigation (equal); methodology (equal); project administration (equal); software (equal); writing – original draft (equal). **Muhammad Ather Nadeem:** Formal analysis (equal); investigation (equal); methodology (equal); project administration (equal); validation (equal); writing – original draft (equal); writing – review and editing (equal). **Alaiha Asif:** Data curation (equal); formal analysis (equal); investigation (equal); methodology (equal); project administration (equal); visualization (equal); writing – original draft (equal); writing – review and editing (equal). **Mehak Fatima:** Data curation (equal); formal analysis (equal); investigation (equal); methodology (equal); project administration (equal). **Muhammad Abdul Rahim:** Data curation (equal); formal analysis (equal); project administration (equal); validation (equal); visualization (equal); writing – original draft (equal). **Chinaza Godswill Awuchi:** Data curation (equal); investigation (equal); methodology (equal); project administration (equal); resources (equal); validation (equal); visualization (equal); writing – review and editing (equal).

## FUNDING INFORMATION

No funding was received for this study.

## CONFLICT OF INTEREST STATEMENT

All authors have no conflict of interest.

## CONSENT FOR PUBLICATION

All authors agreed for publication of this manuscript.

## Data Availability

Even though adequate data have been given in the form of tables, all authors declare that if more data are required then the data will be provided on request basis.
